# Real-Time Prediction of Observed Action Requires Integrity of the Dorsal Premotor Cortex: Evidence From Repetitive Transcranial Magnetic Stimulation

**DOI:** 10.3389/fnhum.2018.00101

**Published:** 2018-03-23

**Authors:** Louisa F. M. Brich, Christine Bächle, Joachim Hermsdörfer, Waltraud Stadler

**Affiliations:** Chair of Human Movement Science, Department of Sport and Health Sciences, Technical University of Munich, Munich, Germany

**Keywords:** action observation, prediction, premotor cortex, simulation, TMS, rTMS, PMd, action occlusion

## Abstract

Studying brain mechanisms underlying the prediction of observed action, the dorsal premotor cortex (PMd) has been suggested a key area. The present study probed this notion using repetitive transcranial magnetic stimulation (rTMS) to test whether interference in this area would affect the accuracy in predicting the time course of object directed actions performed with the right hand. Young and healthy participants observed actions in short videos. These were briefly occluded from view for 600 ms and resumed immediately afterwards. The task was to continue the action mentally and to indicate after each occlusion, whether the action was resumed at the right moment (condition in-time) or shifted. In a first run, single-pulse transcranial magnetic stimulation (sTMS) was delivered over the left primary hand-area during occlusion. In the second run, rTMS over the left PMd was applied during occlusion in half of the participants [experimental group (EG)]. The control group (CG) received sham-rTMS over the same area. Under rTMS, the EG predicted less trials correctly than in the sTMS run. Sham-rTMS in the CG had no effects on prediction. The interference in PMd interacted with the type of manipulation applied to the action’s time course occasionally during occlusion. The performance decrease of the EG was most pronounced in conditions in which the continuations after occlusions were too late in the action’s course. The present results extend earlier findings suggesting that real-time action prediction requires the integrity of the PMd. Different functional roles of this area are discussed. Alternative interpretations consider either simulation of specific motor programming functions or the involvement of a feature-unspecific predictor.

## Introduction

In bodily interactions between individuals, from simple ones such as a handshake to complex and fast ones in sports, action prediction is performed in a temporally highly precise and automatized fashion (Graf et al., 2007; Williams et al., 2011). What information does real-time action prediction require and which mechanisms does it involve? Using repetitive transcranial magnetic stimulation (rTMS) this study aimed to contribute to answering these questions by studying the behavioral consequences of interference in a putative brain structure of action prediction, the dorsal premotor cortex (PMd). This work was following up on an earlier rTMS study in healthy participants (Stadler et al., 2012) which showed weak evidence in favor of a crucial role of the PMd in predicting observed actions during short intervals of occlusion. To replicate and strengthen these results, the current study applied a similar paradigm to a different set of stimuli that showed object directed actions of the right hand and arm. This type of action was chosen, assuming a particular relevance of the PMd for prediction on the kinematic level (spatio-temporal outline) of action (Cavina-Pratesi et al., 2006; Pesaran et al., 2006; Beurze et al., 2007; Filimon et al., 2007). Further, two control conditions were employed which were more conservative than those used in the earlier study (Stadler et al., 2012). First, for a within-subject baseline comparison, the rTMS over the PMd was contrasted to a single-pulse transcranial magnetic stimulation (sTMS) run applied over the primary motor cortex (M1). Second, the effects of rTMS on prediction performance were additionally compared to a control group who received sham-rTMS over the same area.

The idea of the occlusion paradigm is that action prediction enables the participants to detect subtle manipulations of the actions’ course, which are occasionally applied while the action is transiently out of sight. Repetitive TMS delivered over the left PMd during occlusion led to a performance decrement in this task (Stadler et al., 2012). Precisely, the rate of correctly detected shifts in the actions’ time course decreased when rTMS was applied at the onset of occlusions, but not when it started in the second half of the occlusion interval and not under stimulation of a control region (the vertex). This result was taken to suggest a crucial role of the left PMd in real-time action prediction. The involvement of the sensorimotor cortex in this task is in accordance with the action simulation account, suggesting that action prediction and imagery rely on simulation in the observer’s own motor repertoire (Gallese and Goldman, 1998; Jeannerod, 2001; Wolpert et al., 2003; Grush, 2004; Wilson and Knoblich, 2005; Rizzolatti and Sinigaglia, 2010; Avenanti et al., 2013). The relevance of PMd integrity particularly at the onset of occlusions was interpreted as pointing to its role in integrating action-relevant information in the visual scene with motor plans in order to generate a dynamic action sequence internally (Hoshi and Tanji, 2000, 2007; Cisek and Kalaska, 2004; Cavina-Pratesi et al., 2006; Beurze et al., 2007). Also brain imaging studies have associated the premotor cortex with the prediction of observed action (Hesse et al., 2009; Cross et al., 2011; Stadler et al., 2011; Abreu et al., 2012; Balser et al., 2014) and perceptual events in general (Schubotz, 2007; Ptak et al., 2017). Evidence pointing to a major role of the motor system in the prediction and simulation of action was found in several TMS studies. Since the classical findings of neuromuscular modulation through action imagery by Fadiga et al. (1999) and Fadiga et al. (2005), the factors influencing sensorimotor system activation were further determined, suggesting its preferential involvement in not yet completed actions (Urgesi et al., 2006, 2010) and a substantial influence of movement expertise (Aglioti et al., 2008). The effect of expertise was recently corroborated using TMS to induce interference in the PMd which had a detrimental effect on the prediction of the faith of penalty kicks in soccer experts (Makris and Urgesi, 2015). Moreover, rTMS inhibition of the motor system affected the latencies in predictive gaze orientation (Elsner et al., 2013) and the accuracy in predicting action goals was influenced by modulating the ventral premotor cortex (PMv) excitability through non-invasive brain stimulation (Avenanti et al., 2017). Interestingly, also mirror neurons in the monkey homologue of the premotor cortex continued firing during the occlusion of observed grasping (Umilta et al., 2001), pointing to prediction as a general functional principle in the brain network underlying action observation (Kilner et al., 2007; Friston et al., 2011; Donnarumma et al., 2017). In sum, these studies provide evidence in favor of a causal role of motor and premotor areas in action prediction.

However, regarding the specific functional contributions of portions of the sensorimotor system and particularly of the premotor cortex, more data are needed. The effects found in our earlier study (Stadler et al., 2012) were statistically weak. Moreover, the contexts in which action prediction involves either the PMd or the PMv need specification. Assuming multilayered action representation (Grafton and Hamilton, 2007; Friston et al., 2011), predictions on different hierarchical levels might engage different sensorimotor subsystems. For instance, the PMv the inferior frontal gyrus (IFG) seems to be more involved in predicting the goals of actions (Wurm et al., 2014; Avenanti et al., 2017) or object properties (Pobric and Hamilton, 2006; Tidoni et al., 2013), while prediction on the kinematic level involving spatial distances might engage the PMd (Cavina-Pratesi et al., 2006; Pesaran et al., 2006; Beurze et al., 2007; Filimon et al., 2007; Avenanti et al., 2013).

The present study investigated whether interference in the left PMd would affect action prediction on the kinematic level. Two groups of healthy, young participants repeatedly watched six different actions in video clips. The videos showed the right hand and arm performing discrete object directed actions, mostly comparable to prehension performed in studies on the role of the PMd in motor control (Davare et al., 2015). The videos and task design fulfilled some criteria in order to emphasize prediction on a spatio-temporal level which should be clearly distinguished from the level of selecting between multiple action goals. First, due to relatively short occlusions, lasting for 600 ms, prediction covered an interval within a single action step. The internal generation of longer sequences consisting of multiple action steps was not required. Second, the actions were clearly directed toward one single object during the prediction phase. Thus, to succeed in the task, the participants likely predicted the progress on the movement trajectory of the hand that would be achieved after 600 ms of occlusion. After each occlusion, they indicated whether the action continued in time or whether it had not proceeded for the same duration as the occlusion lasted. To detect the time shifts (and discriminate manipulated sequences from those that were in time), the participants were instructed to continue the action mentally during occlusion. The predicted continuation was matched with the action perceived upon occluder offset. The action prediction task was first performed under (sTMS) over M1. In a second run, rTMS (4 supra threshold pulses, 10 Hz) was applied over the left PMd starting at the onset of occlusion. In this run, the experimental group (EG) received effective rTMS and the control group (CG) received sham-rTMS over the same area. The sTMS run was employed as a baseline assuming that it provided a TMS condition under which action prediction was not influenced. The traditional application of sTMS over M1 in the context of action observation, -prediction, and -imagery was to study the activation level in the sensorimotor system through measuring corticospinal excitability (Naish et al., 2014). These designs presumed intact functionality of action prediction and imagery mechanisms during this procedure. Moreover, findings of fMRI studies confirm the absence of M1 contribution but pronounced premotor activation in action prediction and motor imagery tasks that do not require movement execution (Jeannerod, 2001; Hanakawa et al., 2003, 2008; Filimon et al., 2007; Macuga and Frey, 2012). However, there is recent evidence that sTMS over M1 can impair effector representations during action observation (Naish et al., 2016).

We expected detrimental effects of PMd disruption on the prediction accuracy in the EG. This would be expressed in lower rates of trials which are correctly identified as in time or as time-shifted, relative to the sTMS baseline. A comparable impairment in the CG would point to the influence of unspecific factors such as fatigue or general discomfort resulting from the repetitive stimulus on a frontal coil position. Beneficial effects of a repeated exposure to stimuli and task, i.e., learning effects, would lead to increased performance in the rTMS runs of both groups. The results showed that such potential benefits were eliminated by rTMS which induced a decline in prediction accuracy that was exclusively present in the rTMS condition of the EG and was particularly clear in late time shift conditions.

## Materials and Methods

### Participants

Twenty-six healthy participants, between 22 and 34 years of age (mean = 26.88 ± 2.85; 13 male; 13 female), gave written informed consent in accordance with the Declaration of Helsinki. All of them were right-handed according to their own statements. The contraindications of TMS application were excluded by means of a structured questionnaire. None of the participants had a history of neurological or psychiatric disorders. The use of any kind of drugs and medication was negated. The guidelines of the Declaration of Helsinki have been fully complied with (World Medical Association [WMA], 2013) and the protocol was approved by the Ethics committee of the Medical Department at Technical University of Munich. Four participants were later excluded from the statistical analyses due to poor performance expressed in an overall correct rate that was two standard deviations (SD) below the mean. Thus the analyses reported are based on data of 22 participants.

### Stimuli and Task

Six video clips [HD quality, 25 frames per second (frame duration 40 ms), presented on a 32-inch LCD monitor] showed a right-hand and arm performing everyday actions (taking a pen to write on a sheet of paper, putting an espresso cup on a saucer, taking a piece of candy from a bowl, taking a sheet of paper from a pile, taking a clothespin to hang it on a string, taking a tea bag from a cup). The videos were between 4.36 and 7.48 s long (mean 5.62 ± 1.43 s) and were repeatedly presented in randomized order in two task conditions, observation and prediction.

**FIGURE 1:**
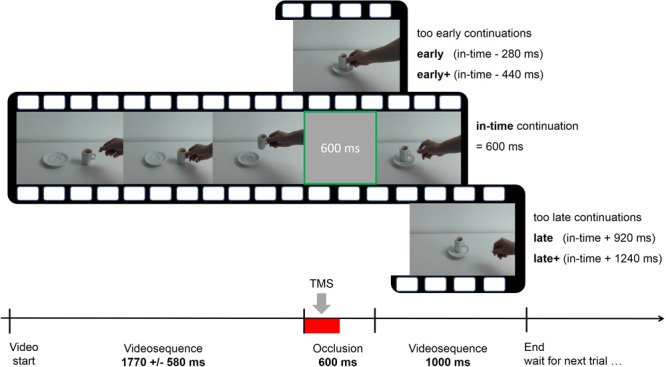
Course of one exemplary trial in the prediction task with five possible time shifts (early+, early, in-time, late, and late+). The videos were occluded for 600 ms and continued immediately afterwards. Single-pulse or repetitive transcranial magnetic stimulation (TMS) was applied during the first 300 ms of the occlusion interval (indicated by the red bar).

In the prediction condition the videos were occluded by a gray screen which was presented between 1 and 2.6 s (mean 1.77 ± 0.58 s) after the beginning of the video and not later than 1 s before the end of the video (**Figure 1**). At the moment of occlusion, the action had evolved until the aim was obvious and the reach- or transport component was initiated. After an occlusion, the video continued for 1 s. In 66% of the trials, the course of the action was manipulated during occlusion. After occlusion, the sequel could thus be either (1) in time (2) not have proceeded enough (action sequence resumed at an event too early in the sequence), and (3) have proceeded too much (action sequence resumed at an event too late in the sequence). The videos in the shifted conditions seemed to have paused during occlusion in the early condition or fast-forwarded in the late condition. In condition “in-time,” the action had advanced for 600 ms, corresponding to the duration of the occlusion (last video frame before occlusion + 15 video frames = action has advanced for 600 ms, given a frame duration of 40 ms). Without making it explicit to the subjects, the early and late conditions were subdivided into two steps. On the early side, the first step had a difference of -280 ms compared to the real-time continuation (“early,” i.e., last frame before occlusion + 7 frames = continuation at 320 ms instead of 600 ms after last observed frame) and the second step differed by -440 ms (“early+”) from the “in-time” condition. In the late condition, two time steps were + 920 ms (“late”) and + 1240 ms (“late+”). This resulted in five time shift conditions (early+, early, in-time, late, and late+) which were randomly presented in each trial, in combination with one of the six actions. The participants were instructed to continue the actions mentally during occlusion, in order to detect disruptions of the actions’ time course. In each trial, they indicated after the occlusion whether the action was continued (1) too early, (2) in-time, or (3) too late. They entered their responses using the ring, middle, and index fingers of the left hand on the respective keys “1,” “2,” and “3” on an external number block.

In the second task, the observation condition, the participants were instructed to watch the actions attentively. Each of the six actions was presented once in random order. To increase attention, one of them was presented twice in succession, resulting in a total of seven trials in this condition. Upon detecting the repetition of an action, the participants were required to press a response key. This task had the purpose to remind them of the full un-manipulated and un-occluded course of the actions.

### Procedure

The 22 participants were assigned to the EG (5 male and 6 female) and the CG (5 male and 6 female). The CG received rTMS over the premotor cortex with a sham coil (sham-rTMS). After a practice phase to familiarize with the experimental procedure and the tasks, the individual motor threshold was determined. Subsequently, the first run of the experiment was performed with sTMS over M1, which was the same for both groups. In addition to the observation and the prediction tasks, the first run of the experiment contained two more conditions, a non-visual action imagery task and an analog to the observation condition showing static geometric figures. These served a secondary research question and are not taken into consideration here. Repetitive TMS or sham-rTMS were always applied in the second run of the experiment. Each run started with the observation condition to remind the participants of the uninterrupted action sequences. In the subsequent prediction task, all six videos were presented about equally often with the five different time shift conditions. The order of presentation and assignment of the six video clips to time shift conditions was pseudo-randomly varied over participants. The prediction conditions in each run (sTMS and rTMS) had both 36 trials. While the in-time condition had 12 trials, the other four time shifts (early+, early, late, and late+) were presented 6 times, resulting in an equal number of responses at each of the three responses buttons (early, in-time, and late). The whole experiment lasted approximately 1–1.5 h.

### TMS

The device PowerMAG 100 research (MAG & More, Munich) was used for the TMS stimulation, with an eight-shaped coil (Double coil PMD70-pCool, pulse duration 160 μS, maximal intensity 2 Tesla) and the sham version of this coil (Double coil PMD70-p-Cool-SHAM), which was visually and haptically identical to the effective coil. This coil had a minimized magnetic field strength, which only enabled the stimulation of the nearest underlying area on the scalp not the brain and produced the same sound and the twitching sensation both varying with intensity as in the effective rTMS coil. The individual resting motor threshold (RMT) was determined using electromyography (EMG; PowerLab, ADInstruments) to record motor-evoked potentials (MEPs) of the right first dorsal interosseous (FDI) muscle through sTMS over the left M1 (hand area C3), according to published criteria (Rossi et al., 2009). For the TMS applications in the experiment, the stimulation intensity was adjusted to 120% of the individual motor threshold, resulting in an average intensity of 55.48 ± 7.21%. In the sTMS condition, the stimulation coil was positioned contralateral to the target muscle over the hand area C3 (extended 10–20 system) 3–4 cm lateral and 1–2 cm anterior to the vertex. The inclination of the coil grip was postero-lateral at a 45° angle to the sagittal axis. Starting from this point, the location of the strongest excitability of the FDI was found (hot spot). In the rTMS and the sham-rTMS conditions, the coils were placed at a distance of 2 cm anterior and 1 cm medial to C3 (Bestmann et al., 2005) with the same inclination of the coil grip. The overlay of the selected position with the left PMd (dorso-caudal edge of the middle frontal gyrus/posterior part of the superior frontal gyrus) was confirmed later using neuronavigation (PowerMAG View!, MAG & More, Munich) with individual high-resolution T1 anatomical brain scans (3T Siemens Verio) in 6 individuals who did not participate in the study. These images with marked target areas were accessible to the reviewers of this paper. In the prediction task of the first run, one single TMS pulse (sTMS run) was delivered randomly during the first 100 ms after the occlusion onset over the hand area in M1. In the second run of the experiment, rTMS or sham-rTMS was applied at the beginning of the occlusion phase over the PMd. Stimulation with four consecutive pulses at 10 Hz started with occlusion onset and lasted for 300 ms.

### Data Analysis

The rate of the correctly answered trials was compared between the different experimental conditions. A total of 22 subjects were included in the statistical analysis, 11 in each group. To study the influence of the five time shift conditions, the correct rates achieved in the sTMS condition were compared to those in the rTMS or sham-rTMS conditions separately for each time shift. For a statistical analysis of the effects of group and time shift, the correct rates of the sTMS condition were subtracted from those achieved under rTMS and a repeated measures mixed ANOVA including the factors time shift (5 levels) and group (EG and CG) was performed on these difference values. Subsequent *post hoc* tests (*t*-tests for independent and dependent samples) served to assess differences between factor levels. Bonferroni correction was applied to account for multiple comparisons. Finally, to determine the distribution of the response “in-time” over the five time shifts, trend analyses were performed separately for the groups and TMS conditions. This was to check whether the in-time responses would follow an expected inverted u-shaped distribution and whether rTMS over the PMd would break up a significant trend. Accordingly, over the course of the five time shifts, good performance would result in a low frequency of in-time responses at early+, a high frequency in the un-shifted condition (truly in-time) and a low frequency at late+.

## Results

Under the influence of rTMS in the EG, prediction performance was impaired relative to sTMS. No such difference was observed in the control group. While the baseline performance in the sTMS runs was similar in both groups (mean correct rate in EG: 66.67 ± 5.12%; mean correct rate in CG: 66.67 ± 10.31%), a performance decline by 9.4% was observed in the EG under rTMS (mean correct rate = 57.32 ± 10.11%). In contrast, the CG achieved a mean correct rate of 70.96 ± 9.65% under sham-rTMS.

**FIGURE 2:**
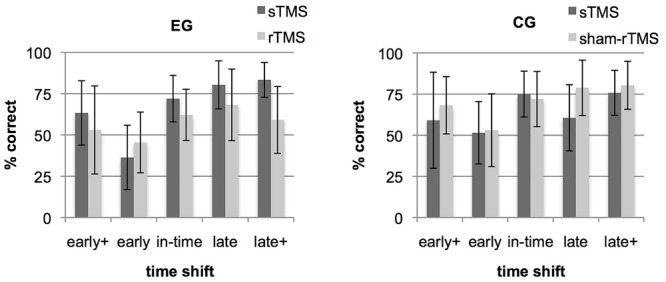
Rate of correct responses separately for each time shift condition (early+, early, in-time, late, and late+). Bars show mean correct rates and SD under single-pulse transcranial magnetic stimulation (sTMS) over M1 (gray) and under repetitive transcranial magnetic stimulation (rTMS) over dorsal premotor cortex (PMd) (black). Separate graphs represent the experimental group (EG) and the control group (CG).

**Figure 2** shows a direct comparison of the correct rates achieved under sTMS and rTMS/sham-rTMS in the different time shift conditions and in each group. Differences in % correct were computed between the sTMS and the rTMS runs for each time shift and group and a repeated measure mixed ANOVA was used to determine the effects of the 5 time shift conditions × 2 groups (effective rTMS in EG, sham-rTMS in CG) on the basis of these difference values. A main effect of group [*F*(1,20) = 17.58, *p* = 0.001, ηp^2^ = 0.47] and an interaction between group and time shift [*F*(4,80) = 2.63, *p* = 0.041, ηp^2^ = 0.12] were found. A main effect of time shift was not significant.

*Post hoc* tests on within-group differences between the five time shift conditions revealed for the EG a significant effect only for the comparison between the conditions early and late [*t*(10) = 4.28; *p* = 0.02]. For the CG, none of the comparisons between all five time shift conditions was significant. For between-group comparisons within each time shift condition, independent samples *t*-tests showed significant group differences for the condition late+ [*t*(20) = -3,63; *p* = 0.01] and a tendency for condition late which did not survive the multiple-comparisons correction [*t*(20) = -2,72; *p* = 0.065]. To demonstrate that these relations are present in the majority of the participants, **Supplementary Figure S1** shows the difference values between rTMS and sTMS at every time shift condition for each participant in the two groups. To conclude, the group × time shift interaction was based on two aspects. Both might results from an asymmetry in the stimuli. First, the EG exhibited a relative performance increase during rTMS in condition early (**Figure 2**) which was more frequently mistaken as being in time in the baseline run (see section “Trend Analyses” below and **Figure 4**). Second, in both late time shift conditions, the distances to the in-time continuation were larger than the distance between in-time and the early conditions (**Figure 1**). This led to maximal correct rates in the baseline run in late time shifts. Under rTMS, participants in the EG did no longer benefit from this more obvious difference to discriminate continuations which were too late from those that were in-time.

**FIGURE 3:**
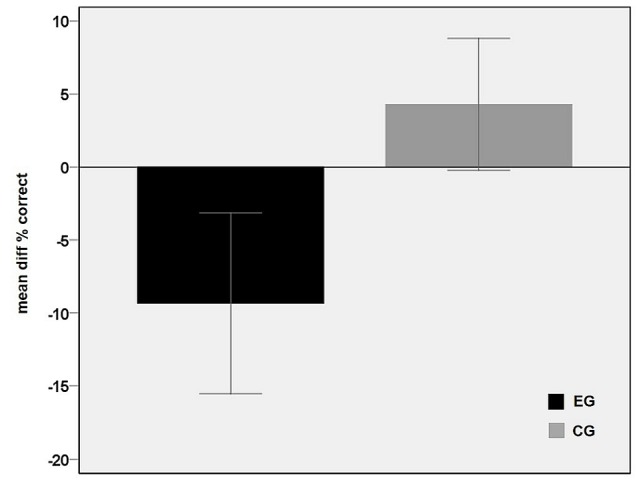
Main effect of group. Differences between the correct rates in the baseline condition (sTMS over M1) and the rTMS condition averaged over the five time shift conditions for each group EG (black) and CG (gray). The negative value in the EG points to a performance decrement during rTMS interference in PMd. Error bars indicate 95% confidence interval.

The main effect of group is shown in **Figure 3** and reflects an overall performance decrease under rTMS in the EG which explains that negative values resulted from the subtraction of the sTMS run from the rTMS run. In contrast, in CG, the correct rates under sham-rTMS were similar or slightly higher than in the single pulse run, leading to difference values above zero (**Figure 3**).

### Trend Analyses

**FIGURE 4:**
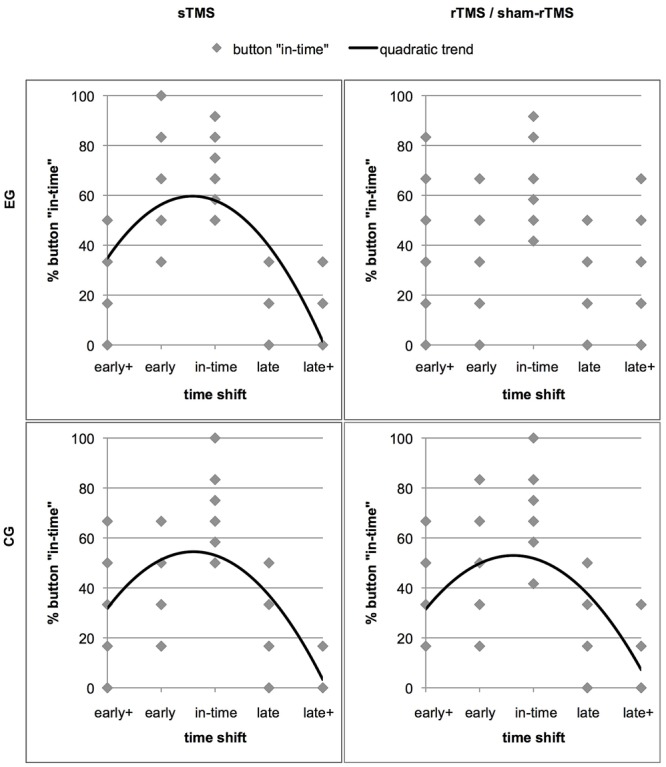
The distribution of the response option “in-time” over the five time shift conditions. The four panels show separate graphs for each stimulation condition (sTMS and rTMS) and each group (effective rTMS in the EG and sham-rTMS in the CG) with significant trend lines. Results of the EG are in the upper panels. The data points represent the rates of “in-time” responses of individual subjects in each time shift condition (Note that the low numbers of points are due to overlay). The better the performance of a group, the more “in-time” responses are given in the un-manipulated time shift condition (“in-time”). This resulted in high percentages of “in-time” responses in the center and low percentages at extreme time shifts (early+ and late+). Less distant shifts (early and late) were more frequently confounded with continuations that were in time. Thus, an inverted u-shaped distribution reflects relatively good performance, which best fits a quadratic trend function in all except the rTMS condition in the EG (upper right panel).

Further insight into how rTMS interfered in the discrimination of shifted from un-shifted (in-time) continuations was obtained by determining the distribution of the response “in-time” over the five time shifts. Relatively good performance should be expressed in an inverted u-shaped distribution with a maximum of “in-time” responses in trials that actually were in time (**Figure 4**). As the stimuli contained an asymmetry between early and late time shifts which was reflected in an asymmetric distribution of in-time responses, we expected a quadratic function instead of a normal distribution to best represent this shape. To test in which conditions this distribution was given, trend analyses including a quadratic function were performed for the sTMS and the rTMS conditions in the two groups. For the sTMS condition of the EG, a significant fit was found (*R*^2^ = 0.522, *F* = 28.43, *p* = 0.000). This reflects the expected distribution of “in-time” responses with a high frequency at the time shift condition that was actually in time and with lowest frequencies at the edges of the timeline. In contrast, no fit was found in the rTMS condition of the EG (*R*^2^ = 0.068, *F* = 1.91, *p* = 0.159). The judgements of action continuations as being in time were less clearly aligned with the un-shifted condition that was truly in time and were given relatively frequently in the other time shift conditions. For the CG, significant results were obtained for both, the sTMS (*R*^2^ = 0.443, *F* = 20.66, *p* = 0.000) and the sham-rTMS (*R*^2^ = 0.364, *F* = 14.89, *p* = 0.000). Thus, also during sham-rTMS, the distribution of the “in-time” responses corresponded to the expected pattern.

## Discussion

The present study tested the relevance of left PMd functions for predicting observed object directed actions performed with the right hand. The use of these stimuli in a paradigm employing brief occlusions after which the action continued immediately stressed prediction on the level of the actions’ spatio-temporal outline. To assess the role of the left PMd in this task, rTMS was applied to interfere in this area during the occlusions. We expected to disturb internal action simulation required for the mental continuation, i.e., prediction, of the action during occlusion (Graf et al., 2007). Indeed, prediction accuracy was significantly reduced under left PMd stimulation which replicated earlier results (Stadler et al., 2012). Overall, this finding points to a relevance of the PMd when spatio-temporal predictions are required. It suggests that the task does not necessarily require action- or goal selection to engage this area. With transient PMd impairment, the EG mistook manipulations of the actions’ time course as correctly timed and vice versa more often than the CG, leading to a significantly lower overall correct rate in the prediction task. The performance decrease relative to baseline levels in EG was particularly pronounced in late time shift conditions, which is discussed in more detail in the following section. With intact PMd in the sTMS run, the participants in both groups were able to identify action continuations that were in time and discriminate them from manipulated trials, which they classified as too early or too late with an accuracy of 67% (**Figure 2**). The higher rate in prediction errors during rTMS in the EG is attributed to the transient absence of the PMd’s contribution to mentally simulate the action. In other words, the results suggest that predicting action dynamics requires the integrity of the PMd.

### Real-Time Action Prediction

As shown in earlier studies employing a similar occlusion paradigm, humans achieve precisely timed predictions of others’ actions (Graf et al., 2007; Parkinson et al., 2012). Here, the relatively low correct rates in the baseline conditions of both groups (mean 66.66%) indicate that the task was not trivial but solvable in most conditions, clearly exceeding the chance level of 33.34% (with three response alternatives). Under rTMS, the overall response rate was significantly lower (57.82%). Taking a closer look to the correct rates in the different time shift conditions (**Figure 3**) and to the distribution of the response option “in-time” (**Figure 4**), more errors were committed in trials with small differences from the correct time courses (early and late) than in extreme shifts (early+ and late+). This pattern is underlined by a maximum of “in-time” responses in the un-shifted condition (truly in time) and a low frequency at the extreme shifts on both sides (early+ and late+). As expected, a significant trend to this inverted u-shaped distribution was confirmed for the sTMS in both groups and for the sham-rTMS in the CG. In contrast, during rTMS the participants confused shifted conditions with the in-time condition more frequently. This yielded a wider dispersion of in-time responses over all time shift conditions and is expressed in the absence of a significant trend. It is further obvious that the early shift was more frequently confounded with in-time than the late one in all conditions (including sTMS baseline and sham-rTMS). This asymmetry between the early and late shifts likely resulted from less physical difference of the early shift to the un-shifted condition (cf. **Figure 1**). It is remarkable though that during rTMS, even the extreme shifts in the late direction (i.e., steps to far into the future), which initially exhibited maximal correct rates, were more frequently judged as being in time. This indicates that with interference in PMd, participants did no longer benefit from larger, more obvious time shifts.

### The Role of the PMd in Action Prediction

Using non-invasive brain stimulation, other authors found evidence in favor of the human premotor cortex as core neural substrate of the prediction of observed action (Pobric and Hamilton, 2006; Tidoni et al., 2013; Makris and Urgesi, 2015; Avenanti et al., 2017). The involvement of motor areas during action observation suggests sensorimotor simulation in the observer’s own motor repertoire to underlie the perception and prediction of others’ actions (Gallese and Goldman, 1998; Jeannerod, 2001; Rizzolatti and Sinigaglia, 2010). The current results corroborate this notion suggesting that similar mechanisms might be involved not only when sensory consequences of one’s own but also when observed action is predicted (Grush, 2004; Davidson and Wolpert, 2005). Predictive functions might be particularly stressed in the absence of visual input, when action prediction, similar to imagery, requires an internally guided transformation of observed or memorized action (Blakemore and Decety, 2001; Blakemore and Sirigu, 2003; Wilson and Knoblich, 2005; Stadler et al., 2011; Ptak et al., 2017). Evidence accumulates for the PMd and interconnected areas [e.g., the superior parietal lobe (SPL)] as core structures driving such internally guided transformations (Cisek and Kalaska, 2004; Abe et al., 2007; Stadler et al., 2011; Ptak et al., 2017).

From the perspective of functional anatomy, a caudal to rostral gradient in the PMd is associated with a shift from strictly motor related functions (PMd proper) to motor-cognitive and visuo-spatial functions (pre-PMd) underlined by a specific connectivity of the different subsections (Boussaoud, 2001; Petrides, 2005; Abe and Hanakawa, 2009; Orban et al., 2015; Genon et al., 2017). Which of the different functions was disrupted in the present study is not easily determined. This is partly due to the distribution of the TMS-induced electric field intensity and to the application method, which were not focal enough for achieving the required selectivity (Bestmann et al., 2005; Bijsterbosch et al., 2012). However, the functional separation between different PMd substructures is also not strict. It rather seems that the proportion of neurons responding to one or the other function gradually changes (Orban et al., 2015). Parallels can be drawn between the “pseudo-hierarchical” (Orban et al., 2015) organization of frontal motor areas and the notion of hierarchies in action representation and motor control (Grafton and Hamilton, 2007; Friston et al., 2011; Ondobaka et al., 2015). The hierarchical level on which action was processed in the present study could point to the required PMd functions. This was determined by the task and the stimulus design. First, the video stimuli showed the right hand and arm interacting with a goal object by approaching, grasping, transporting, or placing it, moving on a trajectory that was defined by the objects’ positions. Second, to discriminate time shifts, the participants needed monitoring the actions’ time course. Both measures guided attention to the dynamics of hand and arm movements and the participants predicted the position on the trajectory that would be reached after 600 ms of occlusion. Regarding the stimulus and task characteristics, it seems plausible that motor programming functions of the PMd were involved to simulate the action dynamics including the initial coding of spatio-temporal parameters such as velocity and acceleration. Indeed, the definition of the movement amplitude (with covariates velocity, acceleration and force) was suggested a function of the contralateral PMd (Churchland et al., 2006; Davare et al., 2015). Alternatively, rTMS might have affected those predictive functions that were suggested to have generalized from an original application in motor control to cognitive tasks (Schubotz, 2007; Ptak et al., 2017). Thus, the observed impairment in prediction might have been due to interference in a task- and stimulus-unspecific prediction or emulation module (Grush, 2004; Ptak et al., 2017). Whether this relies on functions of the caudal or rostral parts or both remains to be clarified. Further, functions on a higher level in the action hierarchy (associated with the rostral PMd) might have been needed in the present task. This involves for instance the integration and maintenance of the behavioral goal (Ondobaka et al., 2015) of the observed person as a precondition, “to step into her shoes” and initiate the simulation of the other person’s action plan (Nakayama et al., 2008).

Other studies found premotor areas outside the PMd, in particular the IFG, to play a role in action prediction. The IFG was associated with the prediction of others’ goals (Wurm et al., 2014; Avenanti et al., 2017) and might for this purpose interact with the posterior parietal cortex, particularly the inferior parietal lobe (Newman-Norlund et al., 2010; Fontana et al., 2012), and the angular gyrus (Hesse et al., 2009; Zwosta et al., 2015). There is also evidence for IFG involvement in prediction on the kinematic level, when observers judged the weight of different boxes from observing someone lifting them (Pobric and Hamilton, 2006; Tidoni et al., 2013). While these studies required integrating the kinematics observed during the hand-object interaction, the contribution of the PMd in the present study might be explained by kinematics covering spatial distances during reach and transport phases (Jeannerod, 1999; Cavina-Pratesi et al., 2006; Pesaran et al., 2006; Beurze et al., 2007; Filimon et al., 2007). The present results are not sufficient to determine the specificity of PMd involvement in action prediction, as none of these additional candidate areas were tested under the same stimulation protocol. Further studies are needed which directly contrast the role of the PMd to that of other candidate areas, the IFG or posterior parietal areas, in the same prediction task.

An alternative interpretation of the present results takes domain-general functions of the PMd into consideration. Instead of affecting action specific processing, rTMS might have interfered in domain general time perception or production. Timing is an integral component of action prediction on the kinematic (i.e., spatio-temporal) level and might be additionally emphasized by the constant duration of the occlusion interval. Several studies highlight the involvement of the PMd when attention is payed to time (Coull et al., 2004), when durations are compared (Rao et al., 2001) or produced, as for instance in sensorimotor synchronization tasks (reviewed in Repp and Su, 2013). A spectrum of other cognitive tasks engages the PMd even in the absense of action. As it is suggested for these tasks, functions relevant for internal timing might have evolved from components of action control during ontogeny (Ptak et al., 2017). To which extent domain-general processes in relation to action-specific functions were affected by rTMS during the prediction of occluded actions can only be speculated here and is another topic which requires further testing.

### Summary

Left PMd integrity was required for successful action prediction when the reach and transport phases of object directed actions performed with the right hand were transiently occluded. This finding replicated and extended earlier studies, which suggested a role for the PMd during the prediction of occluded full body actions (Stadler et al., 2011, 2012). The integration of these studies with the present findings suggests the relevance of the PMd for predicting both, either complex actions in an environment presenting several manipulable objects or single limb kinematics involving a single object. This might point to the contribution of general predictive functions applicable to action on different hierarchical levels. On the other hand, the use of both kinds of stimuli in a real-time prediction task might have emphasized prediction on the kinematic level, drawing on primal motor programming related PMd functions. To address this dissociation, further research employing direct comparisons between prediction on different levels of action is needed to clarify which of these functions relies on the PMd.

## Author Contributions

LFMB, CB, JH, and WS conceived the experiments. LFMB, CB, and WS designed the paradigm. LFMB and CB collected and analyzed the data. LFMB and WS interpreted the data and drafted the manuscript. JH revised the manuscript.

## Conflict of Interest Statement

The authors declare that the research was conducted in the absence of any commercial or financial relationships that could be construed as a potential conflict of interest.

## Supplementary Material

FIGURE S1 Performance of individual participants under rTMS/sham-rTMS relative to baseline. Difference values resulting from the subtraction of the correct rates achieved in the baseline condition [single-pulse transcranial magnetic stimulation (sTMS) over M1] from the correct rates achieved under repetitive transcranial magnetic stimulation (rTMS) over dorsal premotor cortex (PMd). Negative values result from lower correct rates in the rTMS condition. Values at/close to zero indicate no or minimal differences between the baseline and the rTMS conditions. Bars represent 11 participants in the experimental group (EG; black) and 11 participants in the control group (CG; gray). The five different panels show time shift conditions early+, early, in-time, late, and late+.
