# Supplementary figures and images for: Real-Time Prediction of Observed Action Requires Integrity of the Dorsal Premotor Cortex: Evidence From Repetitive Transcranial Magnetic Stimulation
Source: Front Hum Neurosci. 2018 Mar 23;12:101. doi: 10.3389/fnhum.2018.00101 (PMC5876293; doi:10.3389/fnhum.2018.00101)

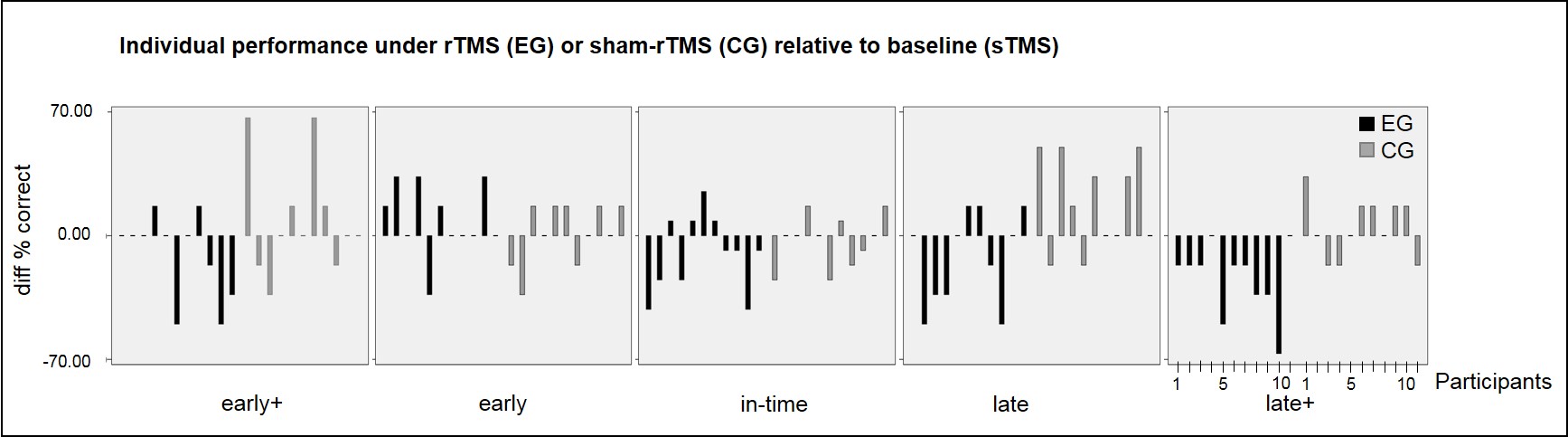

Supplement: FIGURE S1 — Performance of individual participants under rTMS/sham-rTMS relative to baseline. Difference values resulting from the subtraction of the correct rates achieved in the baseline condition [single-pulse transcranial magnetic stimulation (sTMS) over M1] from the correct rates achieved under repetitive transcranial magnetic stimulation (rTMS) over dorsal premotor cortex (PMd). Negative values result from lower correct rates in the rTMS condition. Values at/close to zero indicate no or minimal differences between the baseline and the rTMS conditions. Bars represent 11 participants in the experimental group (EG; black) and 11 participants in the control group (CG; gray). The five different panels show time shift conditions early+, early, in-time, late, and late+. [file Image_1.jpg]
